# Reduction of the P5A-ATPase Spf1p phosphoenzyme by a Ca^2+^-dependent phosphatase

**DOI:** 10.1371/journal.pone.0232476

**Published:** 2020-04-30

**Authors:** Gerardo R. Corradi, Luciana R. Mazzitelli, Guido D. Petrovich, Paula Grenon, Danny M. Sørensen, Michael Palmgren, Felicitas de Tezanos Pinto, Hugo P. Adamo

**Affiliations:** 1 Departamento de Química Biológica, Facultad de Farmacia y Bioquímica, Consejo Nacional de Investigaciones Científicas y Técnicas (CONICET)-Universidad de Buenos Aires, Instituto de Química y Fisicoquímica Biológicas (IQUIFIB), Universidad de Buenos Aires, Buenos Aires, Argentina; 2 Department of Plant and Environmental Sciences, University of Copenhagen, Copenhagen, Denmark; University of Michigan, UNITED STATES

## Abstract

P5 ATPases are eukaryotic pumps important for cellular metal ion, lipid and protein homeostasis; however, their transported substrate, if any, remains to be identified. Ca^2+^ was proposed to act as a ligand of P5 ATPases because it decreases the level of phosphoenzyme of the Spf1p P5A ATPase from *Saccharomyces cerevisiae*. Repeating previous purification protocols, we obtained a purified preparation of Spf1p that was close to homogeneity and exhibited ATP hydrolytic activity that was stimulated by the addition of CaCl_2_. Strikingly, a preparation of a catalytically dead mutant Spf1p (D^487^N) also exhibited Ca^2+^-dependent ATP hydrolytic activity. These results indicated that the Spf1p preparation contained a co-purifying protein capable of hydrolyzing ATP at a high rate. The activity was likely due to a phosphatase, since the protein i) was highly active when pNPP was used as substrate, ii) required Ca^2+^ or Zn^2+^ for activity, and iii) was strongly inhibited by molybdate, beryllium and other phosphatase substrates. Mass spectrometry identified the phosphatase Pho8p as a contaminant of the Spf1p preparation. Modification of the purification procedure led to a contaminant-free Spf1p preparation that was neither stimulated by Ca^2+^ nor inhibited by EGTA or molybdate. The phosphoenzyme levels of a contaminant-free Spf1p preparation were not affected by Ca^2+^. These results indicate that the reported effects of Ca^2+^ on Spf1p do not reflect the intrinsic properties of Spf1p but are mediated by the activity of the accompanying phosphatase.

## Introduction

P5 ATPases are the most intriguing members of the large family of P-type ATPase membrane proteins, which couple ATP hydrolysis with the active transport of ligands across biological membranes [1]. The primary sequence of P5 ATPases suggests that they have a molecular architecture similar to that of other P-type ATPases [2]. However, the molecular function and putative transport activity of P5 ATPases remain unknown.

P5 ATPases are ubiquitous in eukaryotic cells and are important for cellular functions associated with the ER, Golgi, vacuolar-endo-lysosomal system, and mitochondria [3, 4]. P5-ATPases have been classified into subgroups P5A and P5B on the basis of differences in their amino acid sequence, in particular that of the transmembrane segment 4, which in other P-ATPases is part of the transport site [5]. The human genome contains genes encoding one P5A ATPase (ATP13A1) and four P5B ATPases (ATP13A2–A5) [6,7]. Mutations in the ATP13A2 P5ATPases have been associated with a form of juvenile-onset parkinsonism known as Kufor-Rakeb syndrome [8], neuronal ceroid lipofuscinosis (NCL) [9], and a hereditary form of spastic paraplegia (SPG78) [10]. Mutations in ATP13A4 have been implicated in language delay and autism spectrum disorders [11, 12], and more recently alterations in ATP13A3 have been linked to pulmonary arterial hypertension [13]. The unicellular eukaryote *Saccharomyces cerevisiae* contains only two P5 ATPases: Spf1p (Sensitivity to *P**icchia* *f**arinosa* killer toxin), a P5A ATPase [14] homologous to human ATP13A1, and Ypk9p, a P5B ATPase. Spf1p is localized in ER membranes and its knockout causes a pleiotropic phenotype characterized by ER stress, glycoprotein processing defects, abnormal protein targeting, alterations in lipid and sterol content and distribution, and the loss of Ca^2+^ and Mn^2+^ homeostasis [14–21]. Spf1p is required for virulence of human and plant fungi pathogens [22–23].

A distinctive characteristic of P-type ATPases is the coupling of the transport process with the formation and decomposition of an aspartyl-phosphoenzyme (EP) during each transport cycle [24, 25]. In agreement with predictions based on the primary amino acid sequences, all P5A ATPases characterized so far have been shown to hydrolyze ATP and to form an EP phosphoenzyme intermediate [16, 26–28]. While EP formation seems to proceed with Mg^2+^ as the only required ion, lower levels of EP were detected in the presence of Ca^2+^, an observation that was taken to indicate that Spf1p is possibly regulated by Ca^2+^ [26,27]. Intriguingly, Ca^2+^-dependent EP dephosphorylation did not require the endogenous phosphatase activity of the enzyme [26] and the ATPase activity of Spf1p was only marginally affected by Ca^2+^ [16, 19, 28].

Here, we investigated the properties of purified recombinant Spf1p and the basis of the reported effects of Ca^2+^. We found that purified preparations of recombinant His-tagged Spf1p contained trace amounts of a phosphatase that possessed highly active metal ion-dependent ATPase and phosphatase activities. The activity of the accompanying phosphatase readily reduced the levels of Spf1p EP. Optimization of the purification procedure caused the Ca^2+^-stimulated phosphatase activity to vanish, demonstrating that this activity is not an intrinsic property of the Spf1p enzyme.

## Materials and methods

### Chemicals

Polyoxyethylene-10-laurylether (C_12_E_10_), L-α-phosphatidylcholine (P5638), ATP (disodium salt, vanadium-free), SDS, yeast synthetic drop-out media supplement without leucine, yeast nitrogen base without amino acids, dextrose, enzymes, and cofactors were obtained from Sigma. Tryptone and yeast extract were from Difco and the [γ-^32^P]-ATP was from PerkinElmer Life Sciences (Boston, MA). Salts and reagents were of analytical reagent grade.

### Yeast strain and growth media

The initial expression experiments were performed using *S*. *cerevisiae* strain BY4742 (MATα; his3Δ1; leu2 Δ0; lys2 Δ0; ura3Δ0). We subsequently used the BY4742 knockout strain (MATα; his3Δ1; leu2 Δ0; lys2 Δ0; ura3 Δ0; YEL031w::kanMX4), because the expression levels of Spf1p seemed higher in this strain. Both strains were obtained from Euroscarf. Yeast strains were transformed using the LiAc (lithium acetate) method with plasmids described in [19] and [28].

### Standard purification of Spf1p

The His-Spf1p and His-Spf1p (D^487^N) proteins were constitutively expressed in *Saccharomyces cerevisiae* cells as described previously [27, 28]. Yeast cells were transformed with the pMP625 vector containing a Leu^+^ marker, the PMAI promoter, and the cDNA encoding either wild-type His-Spf1p or the His-Spf1p (D^487^N) mutant, both containing a 9XHis tag at the N-terminus [16]. The growth medium contained 6.7% (w/v) yeast-nitrogen base without amino acids (YNB), 0.67% (w/v) complete supplemented medium minus Leu (Leu−), and 2.2% (w/v) dextrose.

Cells collected from 4 L of culture of yeast expressing Spf1p were lysed in a lysis solution containing 50 mM Tris-HCl (pH 7 at 4°C), 130 mM KCl, 250 mM sucrose, 1 mM phenylmethylsulfonyl fluoride, and 5 mM β-mercaptoethanol. The cell pellet was resuspended in 3 volumes of lysis solution and 4 g of glass beads per gram of yeast. Cells were lysed for 1 minute using a bead beater and then cooled on ice for another minute. This procedure was repeated 30 times. Then, the mixture was centrifuged for 10 minutes at 4,080×g at 4°C to remove unbroken cells and the supernatant was centrifuged for 1 h at 100000×g at 4°C to allow membrane precipitation. Total membrane was resuspended in 15 mL of purification buffer containing 50 mM Tris-HCl (pH 7 at 4°C), 20% (v/v) glycerol, 130 mM KCl, 1 mM MgCl_2_, 5 mM β-mercaptoethanol, and 1 mM phenylmethylsulfonyl fluoride, homogenized in a glass homogenizer, and kept at –80°C until purification.

To purify Spf1p, the microsomes were thawed and resuspended in a total volume of 50 mL of purification buffer containing 20 mM MOPS-K (pH 7.4 at 4°C), 20% glycerol, 130 mM KCl, 1 mM MgCl_2_, 5 mM β-mercaptoethanol, and 1 mM phenylmethylsulfonyl fluoride and solubilized at 4°C for 30 min by adding Triton X-100 to a final concentration of 10 mg/mL. Insoluble membranes and proteins were removed by ultracentrifugation for 40 min at 100,000×g at 4°C. Then, 10 mM imidazole was added to the supernatant and it was loaded onto a 1 ml Ni-NTA agarose column (Qiagen) and washed with 90 column volumes of purification buffer containing 0.05% C_12_E_10_ and 50 mM imidazole. Finally, the protein was eluted in purification buffer containing 0.005% C_12_E_10_ and 150 mM imidazole.

### Production of pure Spf1p

Spf1p-His and Spf1p (D^487^N)-His containing 10XHis tags at the C-terminus were obtained using an inducible expression system [19]. Briefly, yeast cells were transformed with the vector pMP4075 containing a His^+^ marker and the cDNA encoding Spf1p under the control of the *GAL1* promoter. The growth medium contained 1.7% (w/v) YNB, 0.67% (w/v) complete supplemented medium minus His (His−), and 2.2% (w/v) dextrose. To induce Spf1p expression, 2 L of cells growing in selective media were pelleted, washed with sterile water, and resuspended in 2 L of induction media containing 2% (w/v) galactose, 1% (w/v) yeast extract, and 2% (w/v) tryptone. Cells were allowed to grow for 20 h at 28°C and then pelleted and stored at –80°C.

The purification started with 2 L of induced yeast culture expressing Spf1p that had been lysed as described above but using lysis solution supplemented with 1 mM EDTA. Microsomes containing Spf1p were washed with this lysis solution and pelleted twice before resuspension in the purification buffer.

The frozen microsomes were solubilized as described above, supplemented with 10 mM imidazole, and loaded onto a 0.5 ml Ni-NTA agarose column (Qiagen). The column was washed with 50 column volumes of purification buffer containing 0.05% (w/v) C_12_E_10_ and 50 mM imidazole plus 50 column volumes of the same solution but with 100 mM imidazole. The protein was eluted in two steps. The first elution with a solution containing 150 mM imidazole contained some Spf1p protein, but still exhibited a low level of contaminant activity. The second elution with 300 mM imidazole contained pure Spf1p and was pooled, aliquoted, and kept in liquid N_2_.

### Protein assay

The Bradford method [29] was used to estimate the protein concentration during the purification procedure. The final concentration was measured by running aliquots of the purified preparations on a 9% acrylamide SDS-PAGE gel according to Laemmli [30]. Bovine serum albumin was used as a standard and the intensity of the Spf1p was compared with the standard after band staining with Coomassie blue.

*Western blotting*. Proteins were electrophoresed on a 9% acrylamide SDS-PAGE gel according to Laemmli [30] and subsequently electrotransferred onto Millipore Immobilon P membranes. Nonspecific binding was blocked by incubating the membranes for 2 h in a solution of 1% nonfat milk, 0.05% Teen-20 and 160mM NaCl. The membranes were incubated for 1 h with monoclonal antibody antiPho8p 1D3A10 (Abcam) and for staining biotinylated anti-mouse IgG and avidin-peroxidase conjugate were used.

### Phosphoenzyme formation

The phosphorylation reaction was performed as previously described [27]. Briefly, 1.5 μg of purified Spf1p was phosphorylated at 4°C in 0.25 mL reaction buffer containing 50 mM Tris–HCl (pH 7.4), 0.5 mM EGTA, 0.5 μM ATP, MgCl_2_ to give a concentration of 2 mM Mg^2+^, and CaCl_2_ to give a final concentration of 10 μM Ca^2+^. Before phosphorylation, 1.5 μg of Spf1p protein was supplemented with 20 μg of C_12_E_10_ and 20 μg of phosphatidylcholine (PC). This suspension was mixed and preincubated for at least 5 min on ice before it was added to the reaction medium. The phosphorylation reaction started with the addition of [γ^32^P]-ATP and was stopped after 15 s with 10% (v/v) ice-cold trichloroacetic acid. The denatured proteins were collected by centrifugation at 20,000×g for 10 min at 4°C and washed once with distilled water. Then, the precipitated protein was suspended in sample buffer and separated by acidic SDS-PAGE. For quantitation, slices of the gel containing the Spf1p EP were cut and the radioactivity measured in a scintillation counter.

### ATPase activity

The ATPase activity was estimated at 28°C based on the release of Pi from ATP using the de Baginski method [31] in a final volume of 0.25 mL of reaction medium containing 50 mM Tris–HCl (pH 7.2 at 28°C), 0.5 mM EGTA, 5 mM N_3_Na, 5 mM MgCl_2_ to give a final concentration of 2 mM Mg^2+^, 3 mM ATP, and 1 μg of Spf1p in 40 μL of elution buffer. When indicated in the text, ZnCl_2_ or CaCl_2_ was added to the reaction media to give a final concentration of 1 μM or 10 μM of the free ions, respectively. Prior to the addition to the reaction medium, Spf1p was supplemented with 40 μg of C_12_E_10_ and 40 μg of PC, and this mixture was preincubated for 5 min on ice. Then, 50 μL of this mixture was transferred to the reaction medium and the sample was incubated for 5 minutes at 28°C prior to the addition of ATP. The enzymatic reaction was stopped after 30 minutes (or the time point indicated in the figure) by the addition of 250 μL of reagent A (0.5% [w/v] ammonium heptamolybdate and 3% [w/v] ascorbic acid in 0.5 N HCl at 4°C). The resulting mixture was incubated on ice for 20 minutes, after which 500 μL of reagent B (2% [w/v] sodium citrate, 2% [w/v] sodium arsenite, and 2% [v/v] acetic acid) were added and the tubes were incubated at 37°C for 20 minutes. After approximately 20 minutes at room temperature, absorbance was recorded at 850 nm.

The inhibitory effect of phosphatase substrates on the contaminant ATPase activity (Fig 3D) was examined at a lower concentration of ATP of 30 μM ATP, closer to that used for the formation of phosphoenzyme. In this case the ATP hydrolysis was estimated as described previously [27] from the release of ^32^P from [γ-^32^P]ATP at 28 °C in a final volume of 0.25 ml of reaction media containing 50 mM Tris–HCl (pH 7.2 at 28°C), 5 mM N_3_Na, 0.5 mM EGTA, 5 mM MgCl_2_, 30μM ATP, 1 μg of Spf1p (D^487^N) in 40 μL of elution buffer, and 0.5 mM of CaCl_2_ to give a final concentration of 10 μM of free Ca^2+^ and the indicated phosphatase substrates. The enzyme was supplemented with 40 μg of C_12_E_10_ and 40 μg of PC and this mixture was then transferred to the reaction media, incubated for 5 minutes at 28°C prior to the addition of ATP to start the reaction. Then, the reaction was stopped after 2 minutes incubation by acid denaturation.

### pNPPase activity

The pNPP hydrolysis was measured at 28°C by following the release of p-nitrophenol from p-nitrophenylphosphate as previously described [32]. The standard mixture used in the pNPP hydrolysis assay was 50 mM Tris-HCl 7.2 at 28°C, 5 mM N_3_Na, and MgCl_2_ and EGTA added to give a final concentration of 5 mM and 0.5 mM, respectively. Prior to the addition of the pNPP to the standard mixture, Spf1p was supplemented with C_12_E_10_ and PC as indicated to quantify ATPase activity. The reaction was carried out for 20 min and stopped by the addition of 1 mL of 1 M NaOH. The tubes were centrifuged at 19,000×g for 5 min at 4°C and the optical density of the supernatants was determined at 410 nm. Blanks obtained without protein were subtracted from each data point. A molar extinction coefficient of 1.78 × 10^4^ M^− 1^cm^− 1^ was used to convert optical density to micromoles of p-nitrophenol released.

### Mass spectrometry

The proteins were separated by SDS-PAGE and stained with Coomassie blue and the colored bands corresponding to Mr 25–116 kDa were cut, destained, and treated with 10 mM DTT for 30 min at room temperature and with 55 mM iodoacetamide in darkness at room temperature. The proteins were digested “in gel” with 25 ng/μL trypsin (Promega, Madison, WI) overnight. The sample was loaded in a μZipTip C18 and eluted with a minimal volume of acetonitrile/matrix 10% mg/mL (α-Cyano-4-hydroxycinnamic acid) onto the MALDI target plate. The extracted tryptic peptides were subjected to concerted MALDI TOF TOF in an ABI4800 mass spectrometer and subsequent MS/MS of the fragments. Peptides were identified by searching the mass profiles of Swiss-Prot using the engine MASCOT (http://www.matrixscience.com).

### Data analysis

The data points are the average of duplicates from representative experiments performed with three to five independent purified protein preparations. Best fitting values of the parameters and their S.E. were obtained by fitting the equations indicated in the legends to the experimental data using SigmaPlot 10 scientific data analysis and graphing software (Systat Software Inc., CA) for Windows.

## Results

### Standard preparations of Spf1p exhibit ATPase activity not related to Spf1p

**Fig 1:**
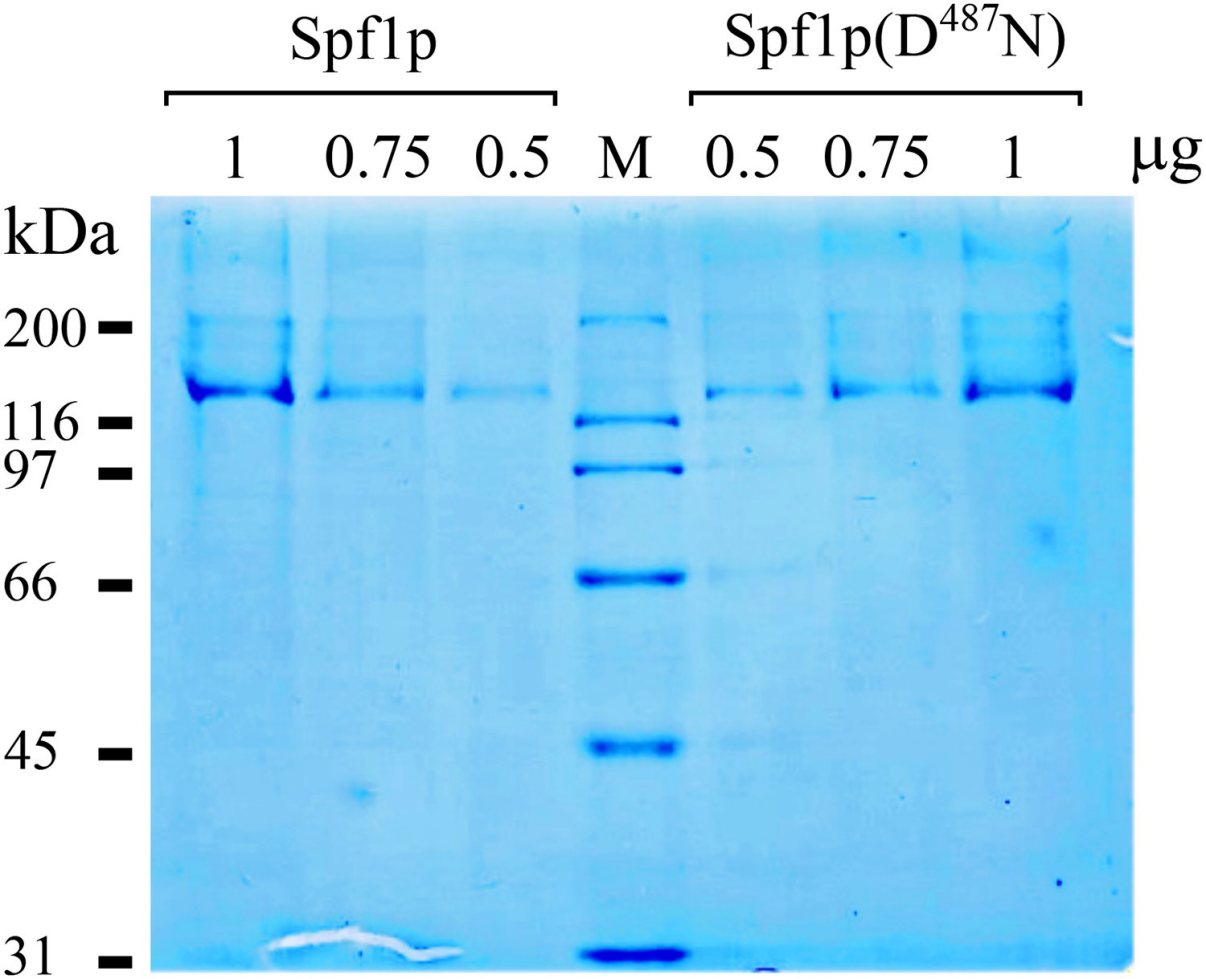
SDS-PAGE of purified Spf1p and Spf1p (D^487^N) preparations. Different volumes of the final 150 mM imidazole eluates of Ni^-^NTA-purified preparations of Spf1p and Spf1p (D^487^N) were loaded in each well of a 9% SDS-PAGE gel and stained with Coomassie Blue. The estimated μg of Spf1p protein loaded is indicated.

**Fig 2:**
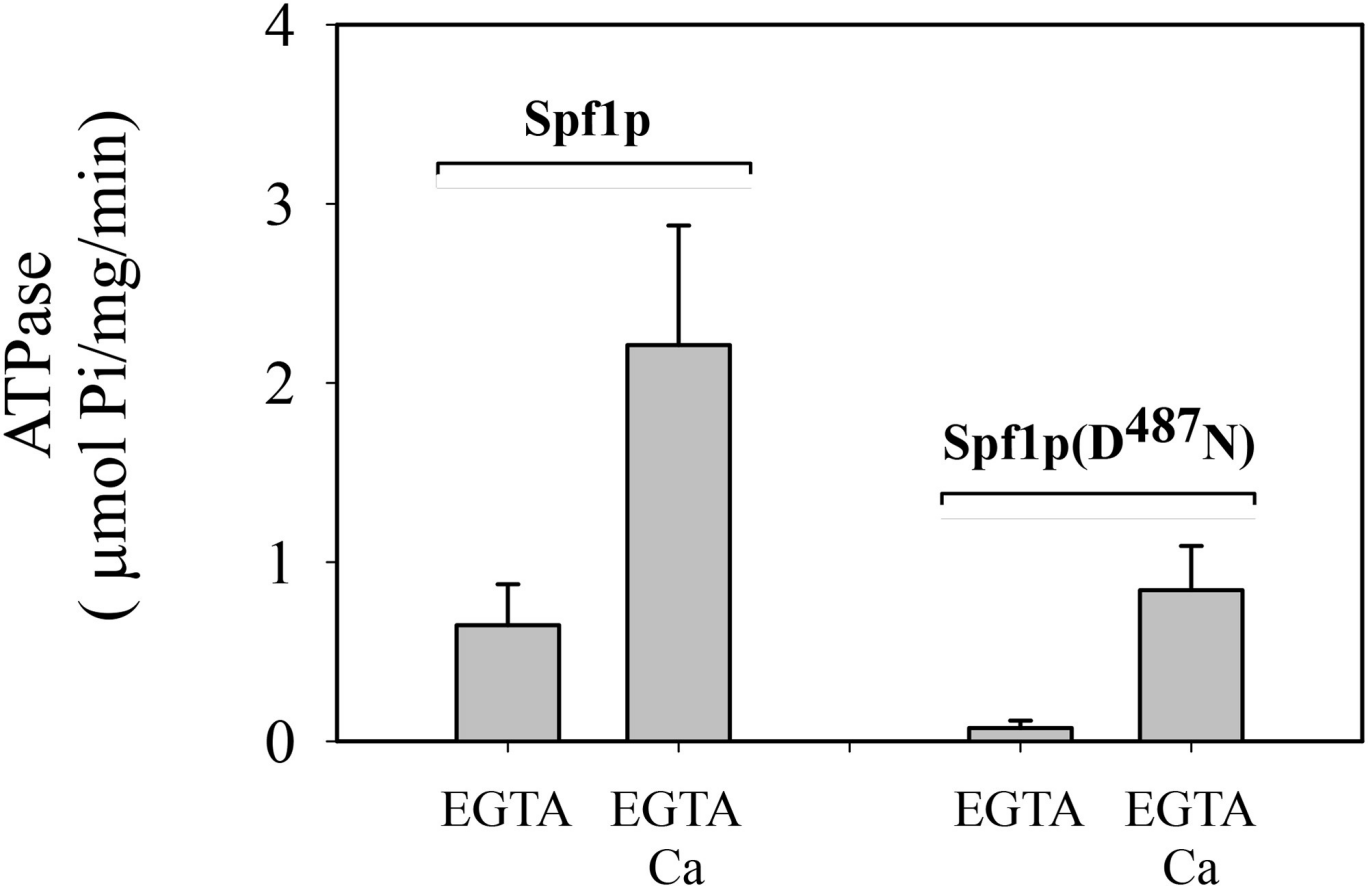
ATP hydrolysis by Spf1p and Spf1p (D^487^N) preparations obtained using the standard purification protocol. The ATPase reaction was performed at 28°C as described in “Materials and Methods” in reaction media containing 50 mM Tris–HCl (pH 7.2 at 28°C), 0.5 mM EGTA, 5 mM N_3_Na, 2 mM Mg^2+^, 3 mM ATP, and 1 μg of Spf1p in 40 μL of elution buffer supplemented with 40 μg of C_12_E_10_ and 40 μg of PC. The reaction time was 20 min. Where indicated, CaCl_2_ was added to the reaction medium to yield 10 μM free Ca^2+^. The data points are from five determinations conducted in duplicate from five independent preparations. *Error bars* show the standard deviation. At the 0.05 level the mean activities in media with EGTA and EGTA-Ca are significantly different.

Recombinant wild-type Spf1p protein and the inactive mutant Spf1p(D^487^N) containing a substitution of the catalytic Asp by Asn were expressed from a constitutive overexpression plasmid in *Saccharomyces cerevisiae* cells, and purified by immobilized metal-affinity chromatography (IMAC) as described in “Materials and Methods” [27, 28]. Both eluates contained mostly a 135-kDa protein on a Coomassie blue-stained SDS-PAGE gel, which is the expected size for Spf1p, and some bands of higher molecular weight, most likely from partially solubilized Spf1p protein (Fig 1). Faint bands with faster migration were also observed when high amounts of protein were loaded. The functional state of the Spf1p purified by the standard protocol was examined by measuring its ATPase activity in a reaction medium containing 0.5 mM EGTA with or without CaCl_2_ to give 10 μM of free Ca^2+^. In the presence of EGTA and the absence of added CaCl_2_, ATP was hydrolyzed at a rate of 0.65 μmol/mg/min **(**Fig 2). The addition of CaCl_2_ to the reaction medium increased the ATPase activity near 3.5 fold. The purified preparation of inactive Spf1p (D^487^N) did not show significant ATP hydrolytic activity in the media with EGTA, but unexpectedly in the presence of CaCl_2_ it hydrolyzed ATP at a rate of 0.90 μmol/mg/min. Thus, the purified preparation of Spf1p contained a contaminating activity stimulated by Ca^2+^.

### The standard preparation of Spf1p is contaminated by an alkaline phosphatase

**Fig 3:**
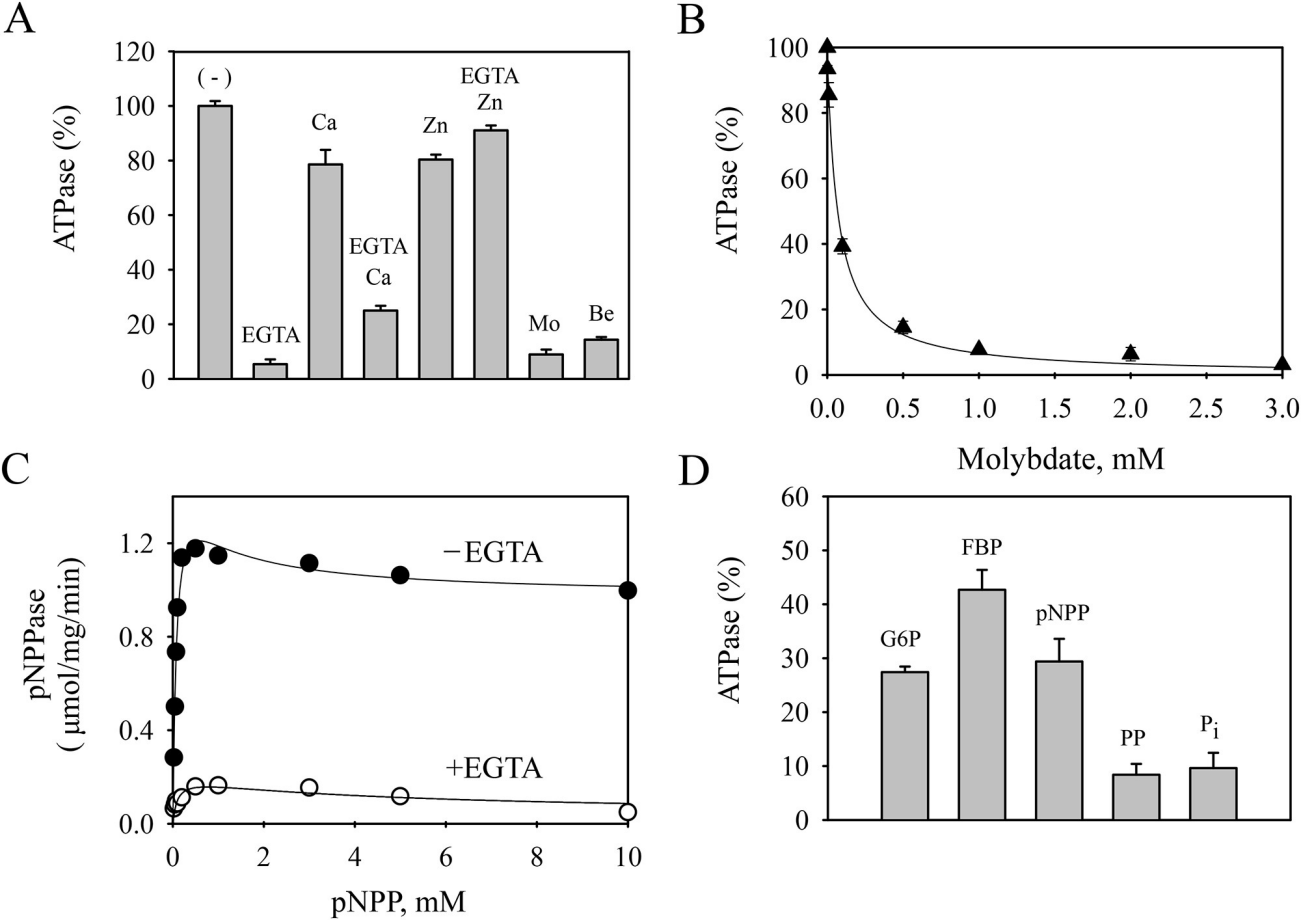
Contaminant ATPase and pNPPase activities in a standard preparation of the inactive mutant Spf1p(D^487^N) obtained by the standard purification protocol. **Panel A-** ATPase activity. The reaction medium was similar to that described in Fig 2 except that EGTA was not present except when indicated. CaCl_2_ and ZnCl_2_ were added to yield 10 μM and 1 μM of the free ions, respectively. Mo, 1 mM ammonium molybdate, Be, 25 μM beryllium sulfate. The ATPase activity of 2.2 μmol/mg/min without additions (-) was taken as 100%. **Panel B-** Effect of increasing concentrations of ammonium molybdate on the contaminant ATPase activity. **Panel C-** pNPPase activity. The hydrolysis of pNPP was measured in the absence or presence of 0.5 mM EGTA. The continuous lines represent fitting to an equation that considers biphasic activation and inhibition components. The K_0.5_ for pNPP of the activation phase was 0.10 ± 0.05 mM for both conditions with and without EGTA. **Panel D-** Inhibition of the contaminant ATPase by phosphatase substrates. The measurement was done as indicated in “Material and Methods” in the presence of 10 μM Ca^2+^. The contaminant ATPase activity at 30 μM ATP concentration was taken as 100%. G6P, glucose 6-phosphate 200 μM, FBP, fructose 1,6 biphosphate 200 μM, pNPP, p-nitrophenyl phosphate 500 μM, PP, phenyl phosphate 500 μM, P_i_, inorganic phosphate 3 mM. The reaction time was 20 min. *Error bars* show the standard deviation.

The contaminant present in the standard Spf1p (D^487^N) preparation had maximal ATPase activity in a reaction medium containing only added Mg^2+^ and was inhibited by EGTA (Fig 3). Ca^2+^ and Zn^2+^ prevented the inhibition produced by EGTA. Beryllium sulfate and ammonium molybdate, commonly used phosphatase inhibitors [33, 34], were highly effective at inhibiting the ATPase of the Spf1p (D^487^N) preparation. Increasing concentrations of molybdate inhibited the ATPase activity of the Spf1p (D^487^N) preparation following a decreasing hyperbola with K_0.5_ ~ 70 μM (Fig 3B). In agreement with the notion that the contaminant could be a phosphatase rather than an ATPase, the contaminant was highly active when pNPP was used as substrate and this activity was also inhibited by EGTA (Fig 3C). Furthermore, various phosphorylated compounds which have previously shown to be phosphatase substrates appear to inhibit the contaminant ATPase activity (Fig 3D).

**Fig 4:**
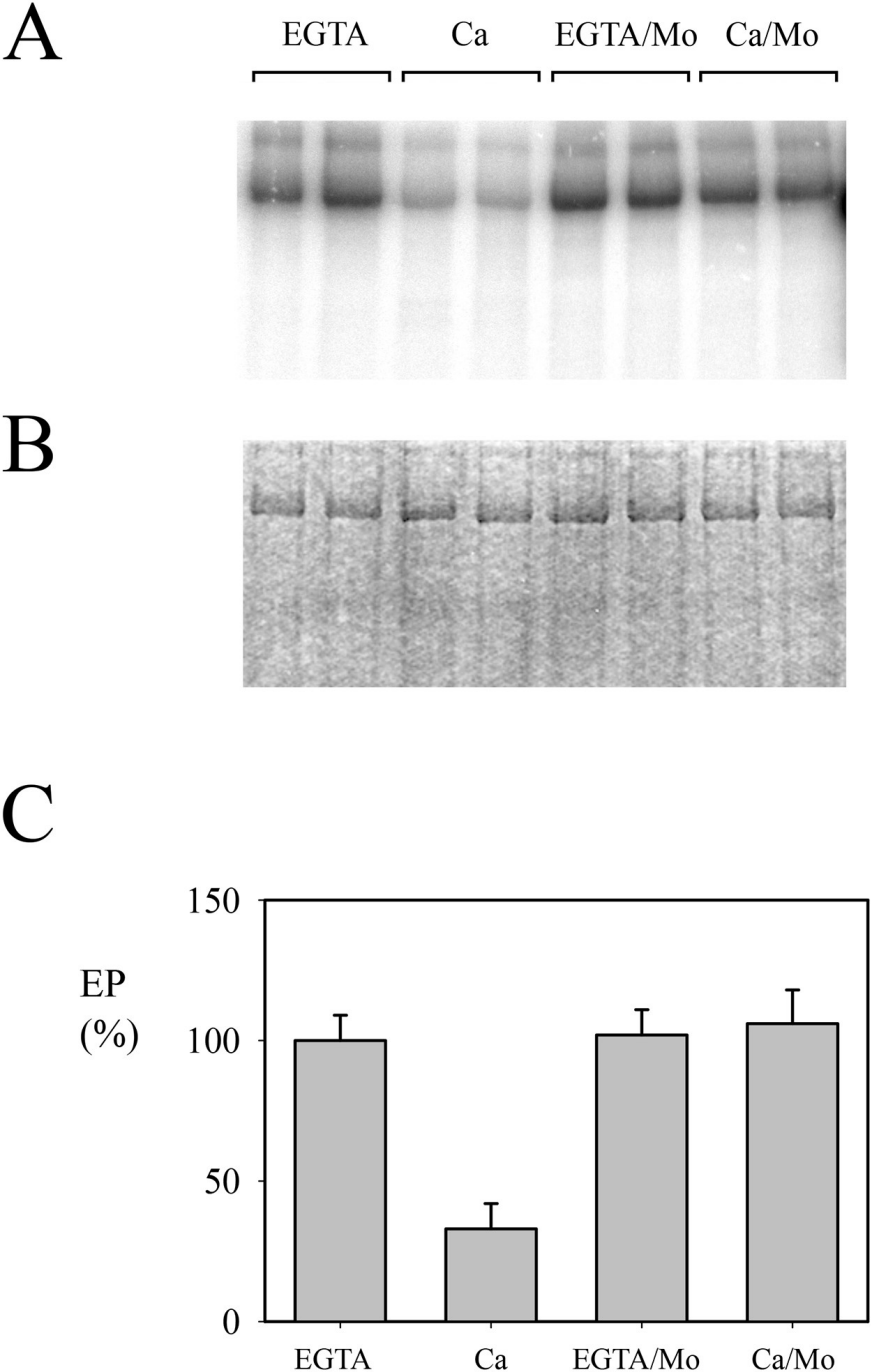
Phosphoenzyme formation by the “contaminated” Spf1p preparation. Spf1p (1.5 μg) was suspended at 4°C in a medium containing 2 mM Mg^2+^, and 0.5 mM EGTA or 0.5 mM EGTA plus enough CaCl_2_ to give a final concentration of 10 μM Ca^2+^. The reaction was started by adding 0.5 μM ATP. Where indicated, 1 mM ammonium molybdate was added to the reaction media. The reaction time was 15 s. **Panel A-** Acidic gel electrophoresis of phosphorylated Spf1p showing the radioactivity. **Panel B-** Coomassie Blue stained gel. **Panel C-** EP levels quantified as described under “Materials and Methods”. Duplicates for each condition are shown. The EP level detected in the EGTA reaction medium was taken as 100%. *Error bars* show the standard deviation.

As previously reported [27], in the standard preparation the level of Spf1p intermediate EP was high when the medium contained EGTA and decreased in the presence of Ca^2+^ (Fig 4). The decrease in the level of EP in “contaminated” preparations of Spf1p persisted even when the concentration of ATP in the phosphorylation media was increased to 100 μM (not shown), suggesting that the lower level of EP was not the simple consequence of ATP consumption. We next examined EP formation in the presence of molybdate, a phosphatase inhibitor. In this condition, the level of EP in the presence of Ca^2+^ increased up to the level observed with EGTA. This result suggested that the EP of Spf1p was dependent on the activity of a contaminating phosphatase.

### Mass spectrometry reveals the presence of contaminating proteins

To determine the identity of the proteins co-purifying with Spf1p in the standard preparation, an aliquot of the Spf1p preparation was resolved by SDS-PAGE and portions of the gel corresponding to a molecular weight of between 25 and 116 kDa were cut and analyzed by mass spectrometry. Peptides from eleven different proteins were identified (Table 1), most of them from membrane-associated proteins with different intracellular localizations. At least five of the identified proteins can hydrolyze ATP. The most abundant protein was Ssa1p, an ATPase involved in protein folding but not reported to be activated by metal ions. Three of them were protein kinases that phosphorylate proteins but do not release Pi (Ck2p, Rtk1p, and Snf1p). The last candidate was Pho8p, a well-characterized alkaline phosphatase that has been reported to dephosphorylate phosphoproteins [35]. This result suggests that the contaminating activity in standard Spf1p preparations was due to the presence of trace amounts of Pho8p.

**Table 1:** Proteins co-purifying with His-Spf1. Protein Mr, intracellular location, median abundance and function was obtained from the *Saccharomyces cerevisiae* Genome Database (SGD). Location: MIT, mitochondria; C, cytosol; N, nucleus; PM, plasma membrane; V, vacuole. Poly-His refers to the presence of a continuous series of at least 6xHis in the primary sequence.

PROTEIN	Mr (kDa)	LOCATION	ABUNDANCE (molecules/cell)	POLY HIS	FUNCTION
POR1 Mitochondrial outer membrane porin 1	30	MIT	21873 ±16504	no	Voltage-gated anion channel; involved in ion transport, cellular redox homeostasis
SRO9, RNA-binding protein	48	C, N	26393 ± 9666	no	RNA-binding protein involved in cytoplasmic translation
yCK2 Casein kinase I homolog2	62	PM	5636 ± 1685	no	Morphogenesis, proper septin assembly, endocytic trafficking, and glucose sensing
PHO8 repressible alkaline phosphatase	63	V	3505 ± 1618	no	Phosphate metabolism, dephosphorylates phosphotyrosyl peptides; contributes to NAD+ metabolism by producing nicotinamide riboside from NMN
SSA1 Heat Shock protein	70	C,N,PM,V	255901 ±159925	no	ATPase involved in protein folding and NLS-directed nuclear transport; member of HSP70 family; required for ubiquitin-dependent degradation of short-lived proteins
RTK1 Probable serine-threonine protein kinase	70	C	3256 ± 1709	yes	Ribosome biogenesis and tRNA synthetase-associated kinase phosphorylated by Cdc28
SNF1 Carbon catabolite-derepressing protein kinase	72	C,N,V,M	5622 ± 2394	yes	AMP-activated protein kinase, required for glucose-repressed gene transcription, heat shock, sporulation, and peroxisome biogenesis
YGR266W uncharacterized protein	81	M, PM	3230 ± 1379	no	Unknown function
PMT4, dolichyl-phosphate-mannose protein mannosyltransferase	88	ER	3728 ± 1570	no	Protein O-mannosyltransferase; transfers mannose residues from dolichyl phosphate-D-mannose to protein serine/threonine residues
CHO2 Phosphatidylethanolamine N methyltransferase	101	ER	7198 ± 3311	no	Conversion of phosphatidylethanolamine to phosphatidylcholine
IST2 increased sodium tolerance protein 2	106	ER	4124 ± 1768	yes	ER-plasma membrane tethering

### Pure Spf1p only exhibits ATPase activity related to Spf1p

To improve the purification procedure, we switched from a constitutive to an inducible expression system, which allowed a higher expression level and a more stringent purification protocol. In addition, we used a variant of Spf1p containing the His tag at its C-terminal end, which was reported to be more efficiently retained by the Ni-NTA column [19]. Moreover, in this purification protocol EDTA was added to the lysis buffer, and microsomes were subjected to more extensive washing before detergent extraction. In addition, a pre-elution step with 50–100 mM imidazole was included to remove contaminant proteins, which may bind weakly to the column before eluting Spf1p with 300 mM imidazole. We named the Spf1p obtained by this protocol “pure” Spf1p.

**Fig 5:**
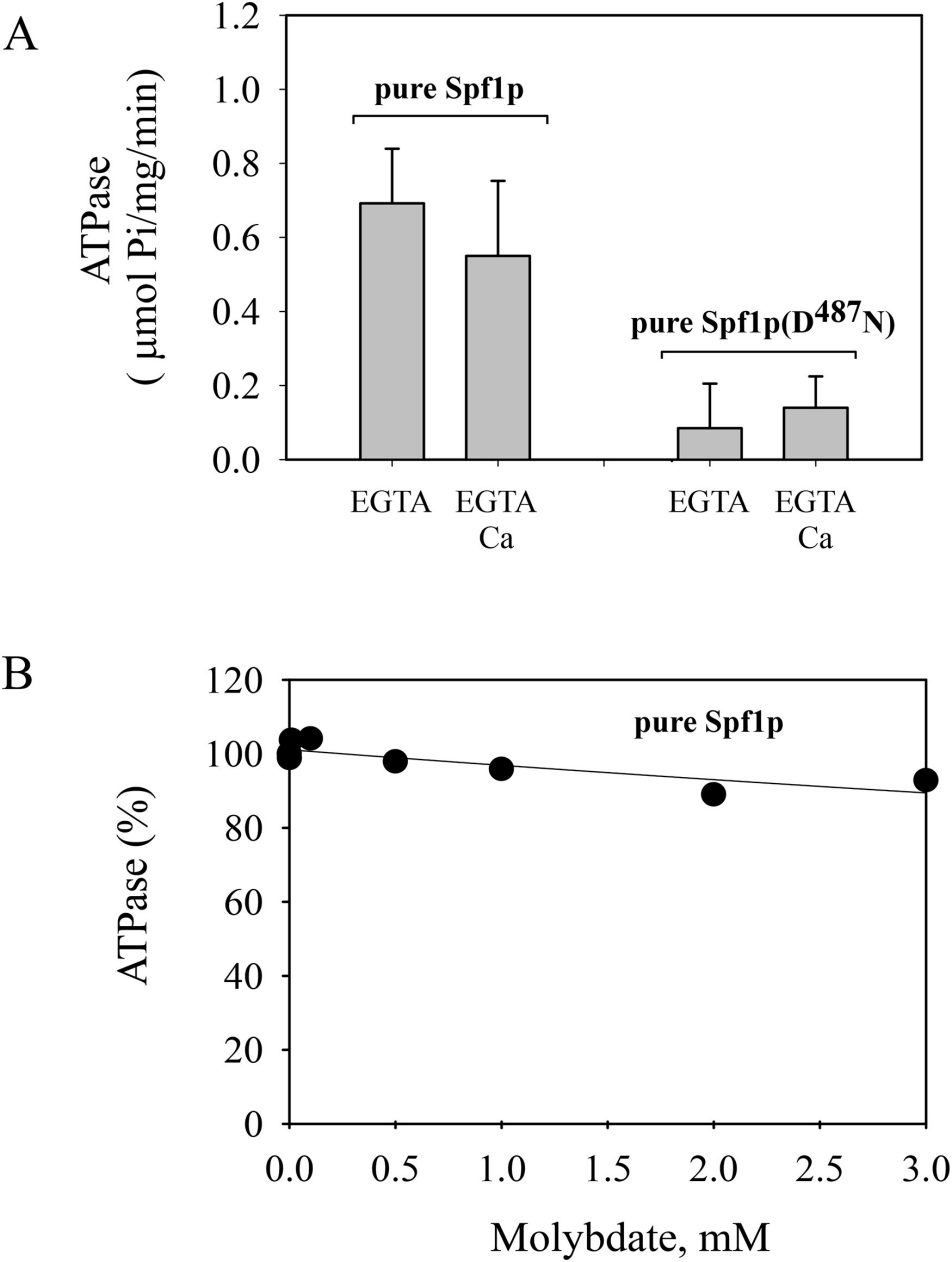
ATPase activity and sensitivity to inhibition by molybdate of the pure Spf1p and Spf1p (D^487^N). **Panel A-** ATP hydrolysis was estimated as indicated in “Materials and Methods”. The composition of the reaction medium was the same as that indicated in Fig 2. The reaction time was 20 min. *Error bars* show the standard deviation. At the 0.05 level the mean activities in media with EGTA and EGTA-Ca are not significantly different. **Panel B-** Effect of increasing concentrations of ammonium molybdate on the ATPase activity of pure Spf1p. The activity at 0 mM molybdate was taken as 100%.

**Fig 6:**
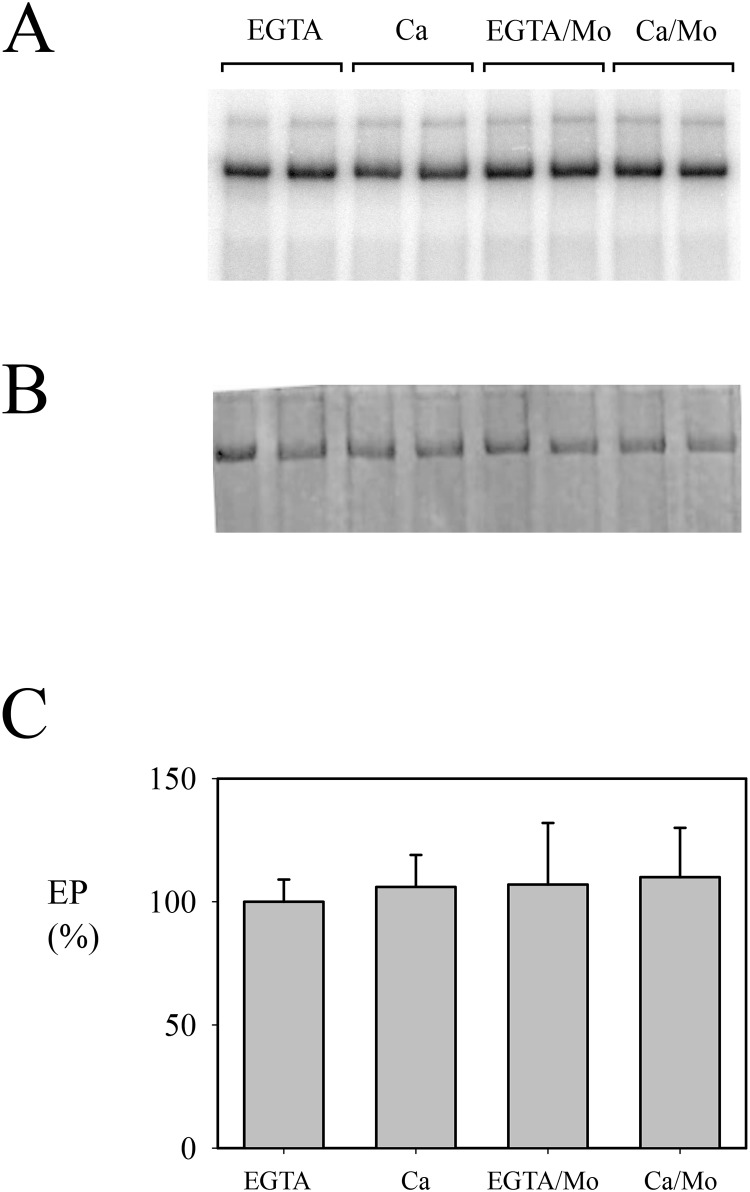
Phosphoenzyme formation by the pure Spf1p preparation. Spf1p (1.5 μg) was phosphorylated under identical conditions as those described in Fig 4. **Panel A-** Acidic gel electrophoresis. **Panel B-** Coomassie Blue-stained gel. **Panel C-** Quantitation of EP. Duplicates for each condition are shown. The EP level detected in the EGTA reaction medium was taken as 100%. *Error bars* show the standard deviation.

**Fig 7:**
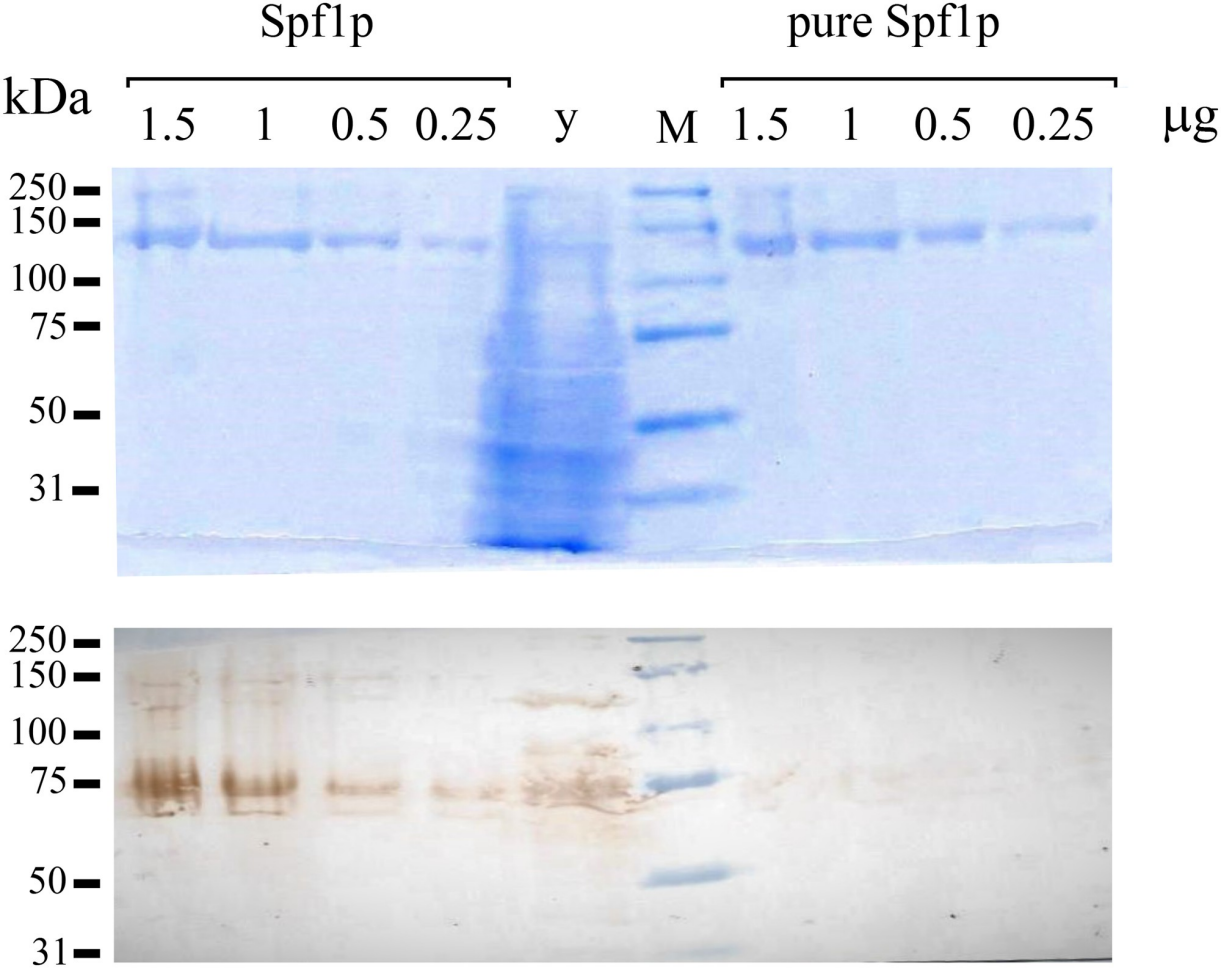
Comparison of Pho8p content of standard and pure Spf1p preparations. *Top*, SDS-PAGE of standard and pure Spf1p preparations. The μg of Spf1p protein loaded in each lane is indicated. M, molecular weight marker, *y*, 20 μg of yeast microsomal protein. *Bottom*, Western blot using antiPho8p antibody.

In a reaction medium containing EGTA, the ATPase activities of pure Spf1p and Spf1p (D^487^N) were similar to those observed in the Spf1p preparations obtained using the standard protocol. However, in contrast with the standard Spf1p preparations, Ca^2+^ did not increase the activity of pure Spf1p, while the pure Spf1p (D^487^N) showed a marginal ATPase activity in the presence of EGTA, and was not stimulated by Ca^2+^ (Fig 5A). Moreover, the ATPase activity of pure Spf1p was unaffected by low concentrations of molybdate (Fig 5B). Notably, the level of EP attained in the pure Spf1p preparation was neither significantly changed by EGTA or Ca^2+^, nor by the addition of molybdate to the phosphorylation media (Fig 6). Together, these observations indicate that the optimized expression and purification protocol successfully removed the contaminant activity from the Spf1p preparation. In line with this idea, we assessed the Pho8p content of the standard and the pure Spf1p preparations by western blot using a specific anti Pho8p antibody. As shown in Fig 7, the Pho8p band was only clearly visible in the standard preparation.

## Discussion

On the basis of indirect evidences, previous studies suggested that Ca^2+^ may be the substrate transported by yeast Spf1p P5A-ATPase. First, a characteristic phenotype of the *SPF1* knockout mutant is the disruption of Ca^2+^ homeostasis and hypersensitivity to external Ca^2+^, reminiscent of the phenotype of a knockout of the Ca^2+^ and Mn^2+^ pump *PMR1* [16]. Second, the levels of Spf1p EP were lower when Ca^2+^ was added to the phosphorylation media, suggesting that Ca^2+^ either inhibited the formation of EP or induced Spf1p dephosphorylation [27]. A similar effect of Ca^2+^ was observed on the homologous plant enzyme HvP5A1 [26]. However, at variance with this hypothesis, in some experiments Ca^2+^ did not seem to produce a significant stimulation of the Spf1p ATPase [16, 19, 28]. This apparent inconsistency led us to investigate in more detail the effect of Ca^2+^ on the Spf1p ATPase.

To characterize Spf1p, we overexpressed recombinant Spf1p and then solubilized the protein with detergents and purified it. Other P-type ATPases have been studied using preparations of isolated membrane or even whole cells. The use of these crude preparations was possible either because specific inhibitors were available (i.e., ouabain for the Na^+^/K^+^-ATPase or thapsigargin for the SERCA pump), or the transported ion could be easily removed from the reaction medium (using metal chelators). None of these conditions are available for any P5 ATPase and hence it is challenging to distinguish its specific activity from the background activity of other cellular ATP hydrolyzing enzymes.

Previously, we used a standard IMAC purification protocol to obtain a preparation of His-tagged Spf1p that, judging by Coomasie blue staining of SDS-PAGE, was more than 95% pure. Using this preparation, we observed that Ca^2+^ inhibited the formation of Spf1p EP [27]. Here, we report that the standard preparation of Spf1p contains low amounts of a phosphatase that can hydrolyze ATP as well as the EP intermediate of Spf1p. This activity requires divalent cations and is inhibited by EGTA. Taken together, the results demonstrate that the reported effects of Ca^2+^ on Spf1p originate from a contaminating phosphatase activity and not from Spf1p itself. The presence of this contaminant may explain the reported apparent spontaneous dephosphorylation of the plant Spf1p analog HvP5A1 [26]. In contrast the contaminant may not have been detected in [16] since Spf1p was purified from sucrose fractionated-ER membranes rather than from total yeast microsomes.

Because the contaminant hydrolyzed not only ATP but also non-nucleotide phosphates such as pNPP, and was inhibited by beryllium and molybdate, we tentatively identified the co-purifying contaminant protein as a phosphatase that is also capable of ATP hydrolysis. Mass spectrometry analysis revealed that three protein kinases, a chaperone, and the phosphatase Pho8p co-purified with Spf1p. The catalytic properties of the contaminating phosphatase fit with that of the well-characterized Pho8p. Pho8p is a repressible non-specific alkaline phosphatase that can act on a variety of phosphorylated compounds [36]. Most strikingly, Pho8p has been reported to dephosphorylate phosphoproteins [32] and is likely that also acts on protein aspartyl phosphates. It therefore seems that Pho8p is the contaminant we have identified in the standard Spf1p preparation. While, other alkaline phosphatases have been shown to exhibit a Ca-dependent ATP hydrolysis activity [37], detailed studies of the effect of Ca^2+^ on the Pho8p activity are not available at present.

Pho8p is a single transmembrane protein that is initially synthesized in the ER and is glycosylated as it moves from the ER to the Golgi. Finally, Pho8p localizes in the vacuolar membrane where it becomes activated by the removal of a C-terminal inhibitory peptide [38]. Pho8p and Spf1p possibly interact during the detergent treatment used to solubilize membrane proteins and co-purify during the Spf1p purification procedure. However, an active soluble Pho8Δ62 truncated form has been described in yeast extracts and hence it may interact with the Spf1p in the cytosol [39].

Our results suggest that a small amount of this highly active Pho8p phosphatase is capable of dephosphorylate Spf1 EP. Although at this stage we have no clues about the molecular mechanism involved, it seems interesting to note that a similar effect of acylphosphatase on the EP of the Na^+^/K^+^-ATPase, the plasma membrane and sarcoplasmic reticulum Ca^2+^ pumps has been described, leading to a higher EP turnover and a lower coupling between transport activity and ATP hydrolysis [40–42].

In summary, the results presented in this paper indicate that Ca^2+^ does not directly stimulate the activity of Spf1p and the previously reported effect of Ca^2+^ on Spf1p EP reflects the action of an exogenous phosphatase, which may be involved in the regulation of Spf1p.

## Supporting information

S1 Raw images (PDF)

## Abbreviations

C_12_E_10_: polyoxyethylene 10-lauryl ether
EP: phosphorylated enzyme
ER: endoplasmic reticulum
HEPES: 4-(2 –hydroxyethyl)-1-piperazine ethane sulfonic acid
IMAC: immobilized metal affinity chromatography
MOPS: 3-(N-morpholino) propanesulfonic acid
MS: mass spectrometry
PC: phosphatidylcholine
pNPP: p-nitrophenylphosphate
SDS-PAGE: sodium dodecyl sulfate-polyacrylamide gel electrophoresis
Spf1p: sensitivity to *Pichia farinosa* killer toxin protein
