# Supplementary material for: Reduction of the P5A-ATPase Spf1p phosphoenzyme by a Ca2+-dependent phosphatase
Source: PLoS One. 2020 Apr 30;15(4):e0232476. doi: 10.1371/journal.pone.0232476 (PMC7192388; doi:10.1371/journal.pone.0232476)
Supplement: S1 Raw images — (PDF) [file pone.0232476.s001.pdf]

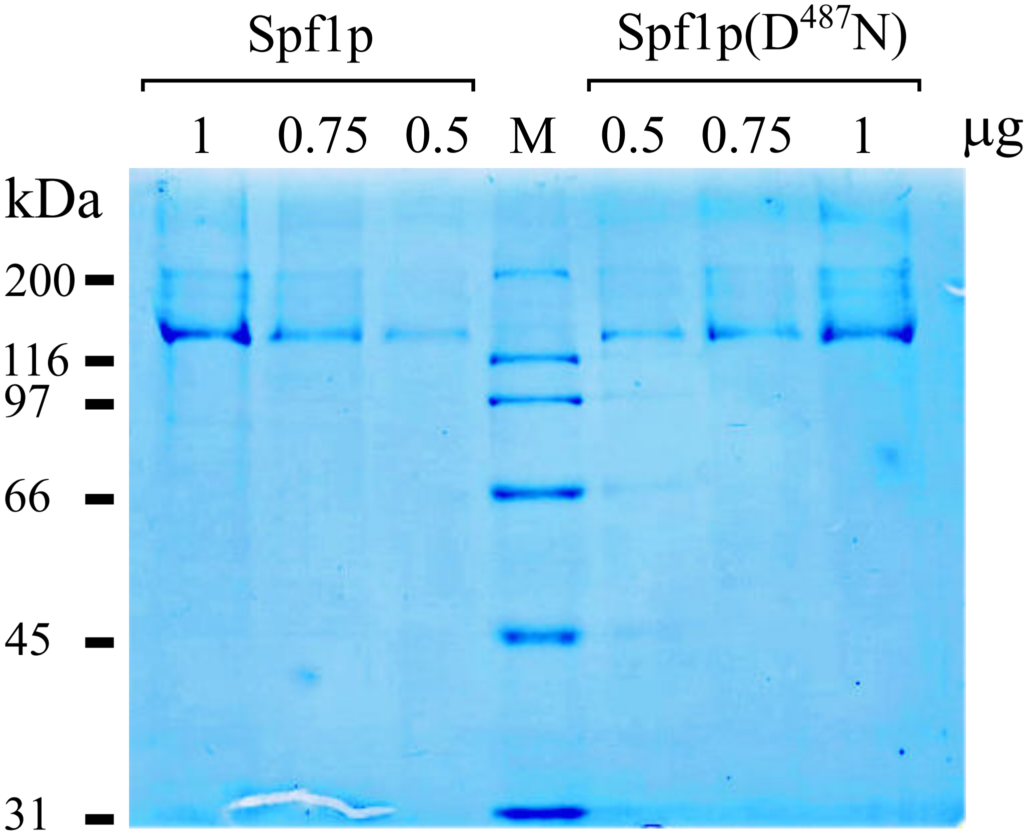

Spf1p
Spf1p(D487N)
1
0.75
0.5
M
0.5
0.75
1
μg
kDa
200
116
97
66
45
31

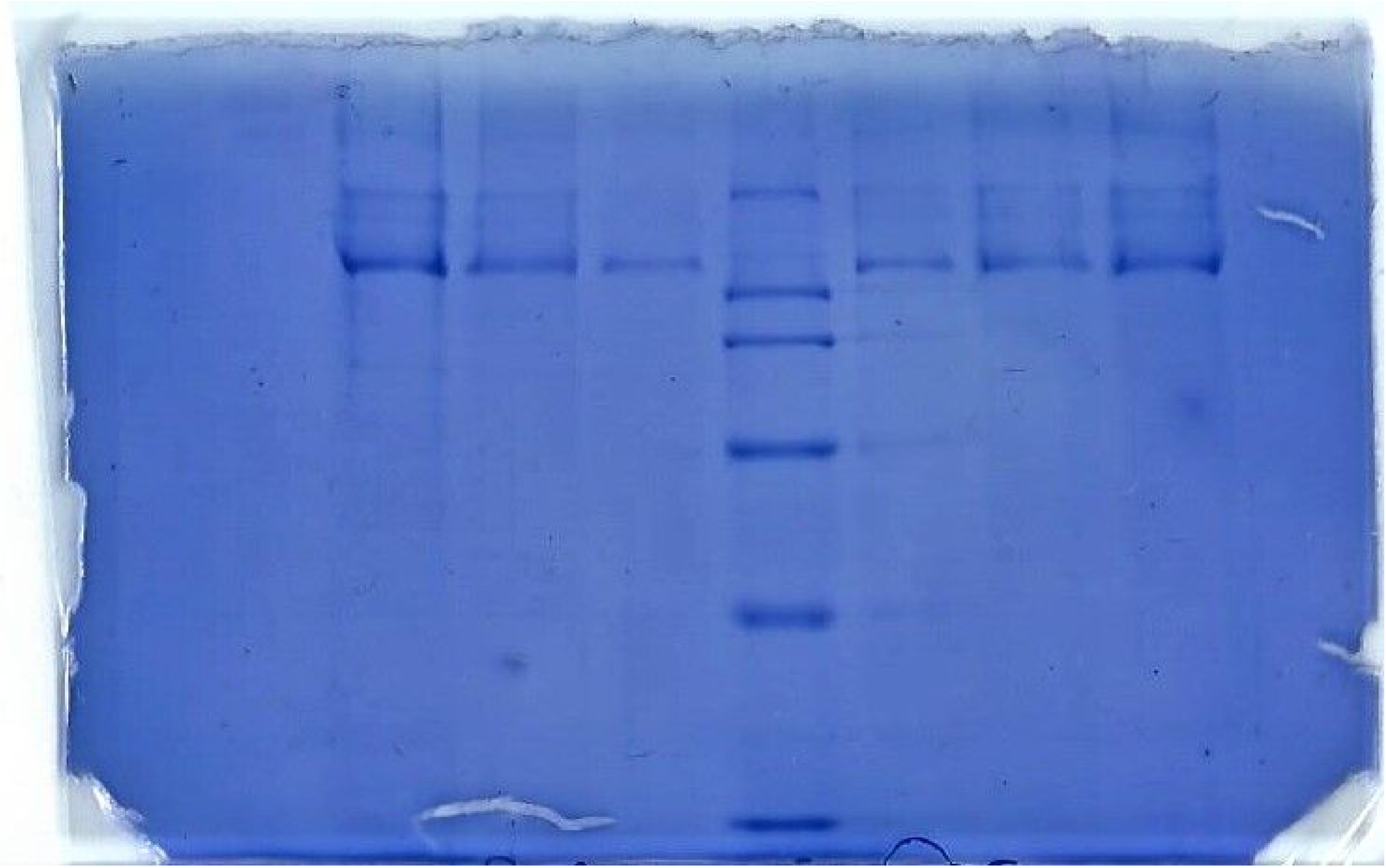

figure 1.tiff

Figure 4

A

B

C

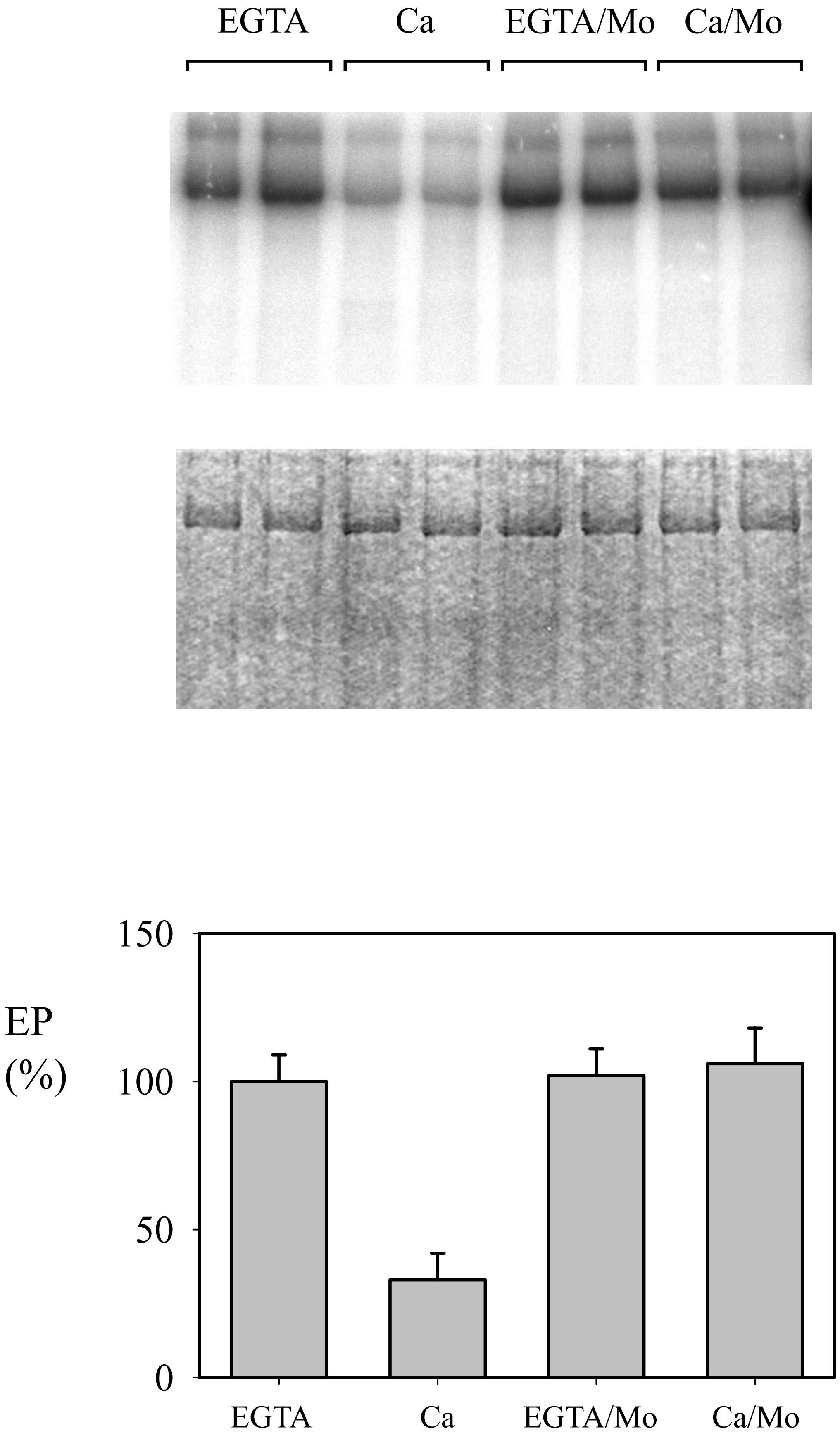

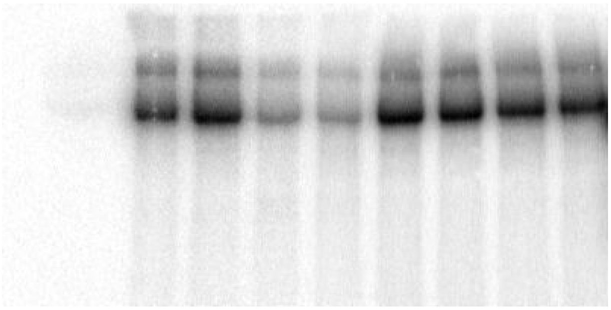

figure 4a.tif

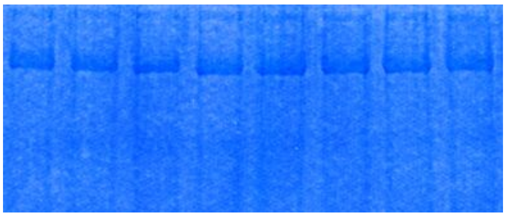

figure 4b.tif

Figure 6

A

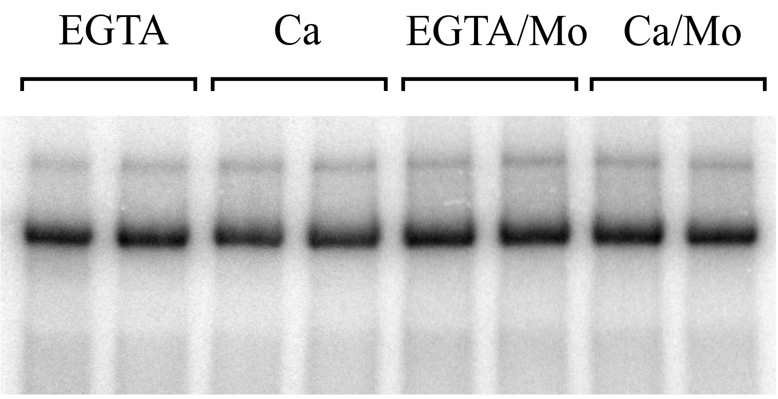

B

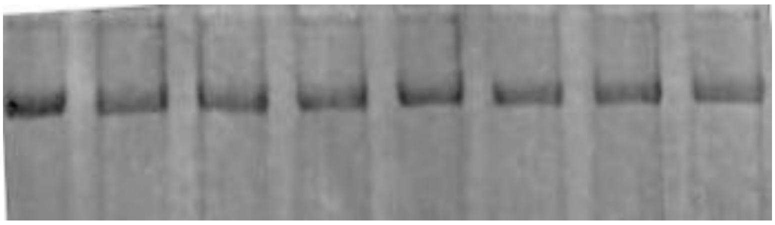

C

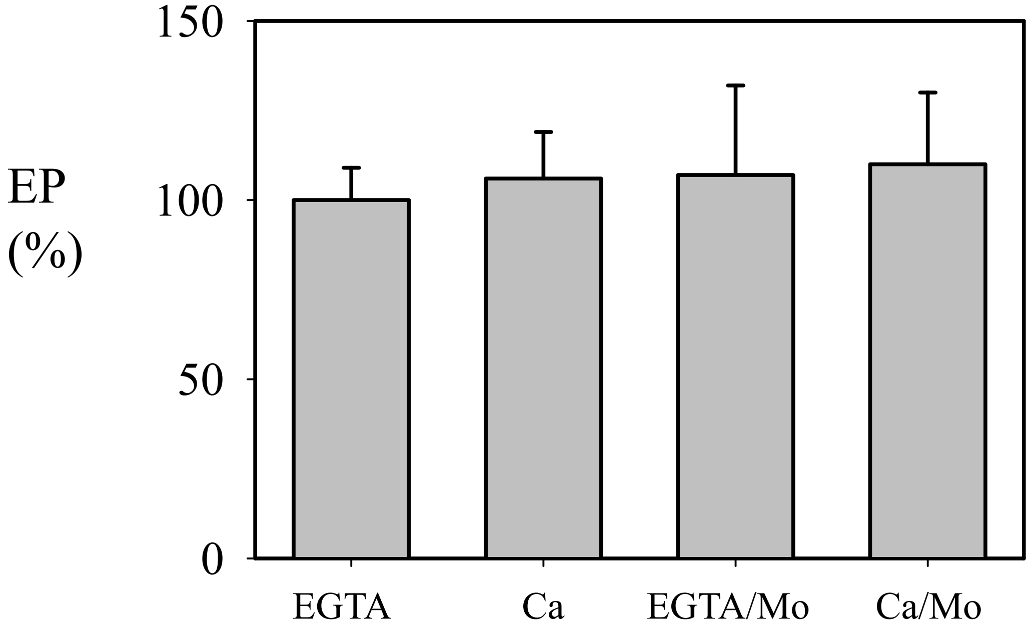

F6.pdf

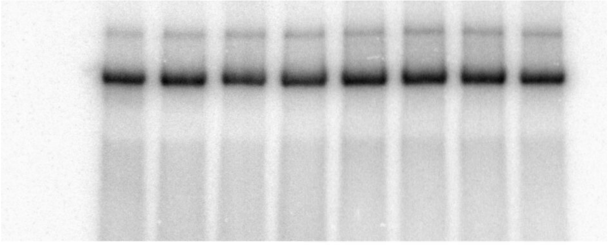

figure 6a.tif

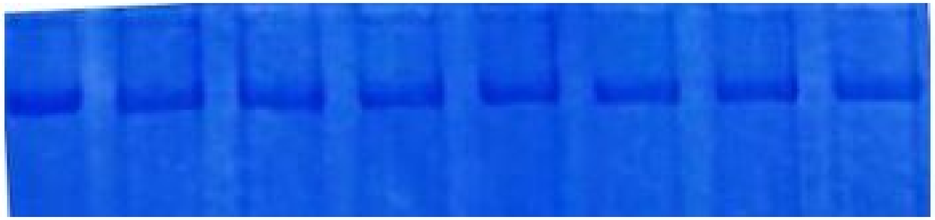

figure 6b.tif

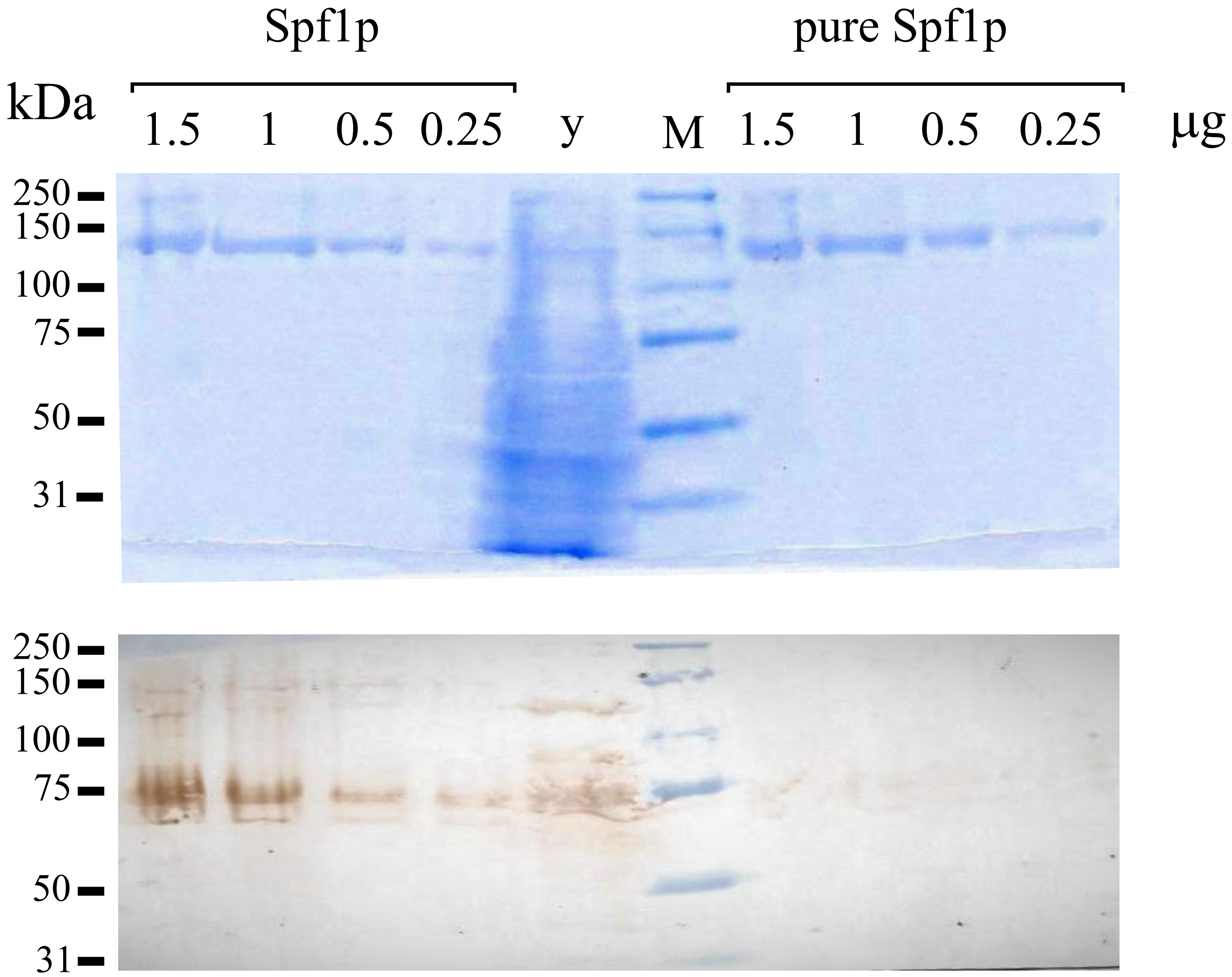
Spf1p
pure Spf1p
kDa
1.5
1
0.5
0.25
y
M
1.5
1
0.5
0.25
µg
250
150
100
75
50
31
250
150
100
75
50
31

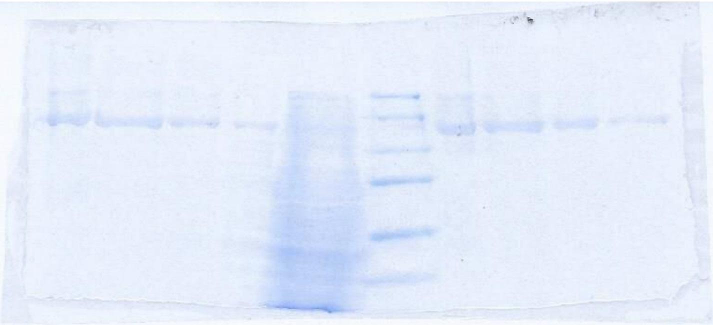

figure 7a.tif

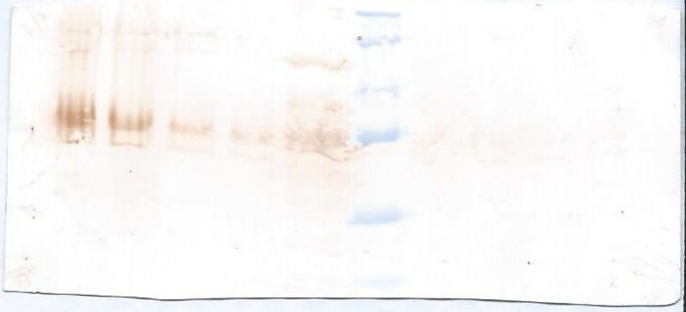

figure 7b.tif
